# Human-centered digital twins in hospitality: how employee perceptions and system design shape adoption

**DOI:** 10.3389/frobt.2026.1772854

**Published:** 2026-04-01

**Authors:** Desiree Manzano-Farray, Moises Segura-Cedres, Lidia Aguiar-Castillo, Maria Jerez-Jerez, Rafael Perez-Jimenez

**Affiliations:** 1 Instituto para el Desarrollo Tecnológico y la Innovación en Comunicaciones (IDeTIC), Universidad de Las Palmas de Gran Canaria, Las Palmas de Gran Canaria, Spain; 2 University of Bedfordshire, Luton, United Kingdom

**Keywords:** digital twin, effort expectancy, gamification, intention to use, performance expectancy, technology adoption, trust in the system

## Abstract

**Introduction:**

The digital transformation of hospitality is increasingly driven by technologies that integrate human and operational elements of service work. Within this evolution, human-centered Digital Twins leverage both human-related and operational data to digitally represent employees within their work contexts, enabling real-time feedback and data-informed decision making for both employees and organizations. Despite their potential, little is known about how hospitality employees perceive these systems or what shapes their willingness to use them.

**Methods:**

This study examines the individual perceptual factors that influence employees’ intention to use a human-centered Digital Twin, focusing on performance expectancy, effort expectancy, and trust in the system. In addition, the study explores the role of gamification as a system design feature that may shape how these perceptions translate into adoption intentions. Data were collected from 141 customer-facing hotel employees across Europe using a structured survey based on validated scales. An Exploratory Factor Analysis confirmed the reliability and structural validity of the measurement model, and multiple linear regression analysis was used to test both the baseline and the extended models.

**Results:**

Results show that all three perceptual factors significantly and positively influence intention to use, with performance expectancy emerging as the strongest predictor. Gamification moderates the relationship between effort expectancy and intention to use in a non-reinforcing manner: when gamification is higher, the positive effect of effort expectancy becomes weaker.

**Discussion:**

These findings suggest that interaction design can alter how employees experience the ease of using advanced digital systems. This study provides empirical evidence on the perceptual determinants that influence front-line employees’ intention to use a human-centered Digital Twin in hospitality settings, highlighting the role of both core adoption beliefs and system design features in shaping adoption intentions.

## 1 Introduction

The relationship between technological development and work has evolved alongside successive waves of digital transformation, with recent research highlighting how contemporary technologies are reshaping tasks, decision-making processes, and human–technology interaction in organizational settings (Baiyere et al., 2020; Vial, 2019). In parallel, management and information systems research has emphasized a shift from purely automation-oriented technologies toward systems designed to augment human work, underscoring the growing importance of human-centered and experience-oriented perspectives (Raisch and Krakowski, 2021). Within this broader evolution, advances in data analytics and artificial intelligence have enabled increasingly adaptive and interactive systems, reinforcing the need to understand how employees perceive and engage with data-driven technologies in real work contexts (Bankins et al., 2024; Lee et al., 2023).

Against this background, understanding how employees adopt emerging data-driven technologies becomes particularly relevant. While classical technology adoption models have been widely applied to explain users’ acceptance of information systems, recent research highlights that data-driven and AI-enabled systems introduce characteristics that challenge traditional assumptions of technology use (Benbya et al., 2020; Lee et al., 2023). Systems such as Digital Twins (DTs) increasingly rely on predictive analytics, algorithmic recommendations, and continuous data processing to enable real-time synchronization and feedback between physical and digital representations (Fuller et al., 2020; Tao et al., 2019). These features can amplify uncertainty, perceived risk, and users’ dependence on system outputs (Bankins et al., 2024; Benbya et al., 2020; Rai et al., 2019). In these contexts, user adoption is shaped not only by expected performance gains, but also by how individuals experience system complexity, cognitive effort, and trust in algorithmic decision support (Bankins et al., 2024; Glikson and Woolley, 2020; Lee et al., 2023). Consequently, contemporary information systems and organizational research emphasize the need to contextualize established adoption frameworks when examining human-technology interaction in socio-technical environments, particularly when technologies directly influence work practices and decision making (Bankins et al., 2024; Benbya et al., 2020; Rai et al., 2019).

Within tourism and hospitality, these technological developments intersect with service environments where human interaction plays a central role in value creation (Buhalis, 2020; Gretzel et al., 2015). Frontline employees are directly involved in service delivery and operational decision making, making their interaction with digital systems particularly critical for organizational performance (Bankins et al., 2024; Lee et al., 2023). In this context, Human Digital Twins (HDTs) have recently emerged as an extension of DT technologies, aiming to create dynamic digital representations of employees that integrate real-time data about work activities, behaviors, and performance indicators (Lin et al., 2024; Yang et al., 2024). Rather than replacing human judgment, such systems are designed to support decision making and enhance employee capabilities within complex service environments (Bankins et al., 2024; Lee et al., 2023; Raisch and Krakowski, 2021).

Despite the growing conceptual interest in DTs and the recent emergence of HDTs, empirical research examining their adoption from the perspective of frontline employees remains scarce, particularly in tourism and hospitality contexts. Existing studies on DTs have primarily focused on technical architectures, operational efficiencies, or customer-facing applications, while research on employee adoption of advanced, data-driven systems has largely examined more generic information technologies. As a result, little is known about how hospitality employees evaluate and respond to a human-centered Digital Twin that continuously represents their own work activities and performance. This study addresses this gap by empirically examining the perceptual determinants of frontline employees’ intention to use a human-centered Digital Twin in hospitality, while also exploring the moderating role of gamification as a design layer within the adoption process.

To achieve this objective, the remainder of the paper is organized as follows. Section 2 reviews the relevant literature and develops the conceptual framework and hypotheses. Section 3 describes the research methodology, including the study design, data collection process, and measurement validation procedures. Section 4 presents the empirical results, while Section 5 discusses and interprets the study’s findings. Finally, Section 6 outlines the theoretical and practical implications of the study, and Section 7 concludes by presenting suggestions for future research together with the main study limitations.

## 2 Literature review

### 2.1 From digital twins to Human Digital Twins

**FIGURE 1:**
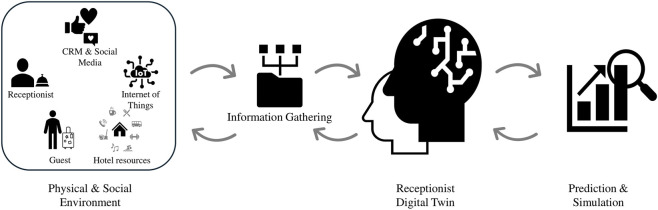
Human-centered Digital Twin model applied to hotel front-line employees.

The rapid digitalization of service environments has driven the development of technologies that connect physical systems with virtual representations supported by continuous data flows. Within this broader shift toward data-driven and human-centered technologies, DTs have emerged as a prominent example of systems that integrate real-time data, analytics, and human interaction within organizational settings (Iliuţă et al., 2024). Thereby, DT has become a key tool because it enables a virtual model to mirror the real-time conditions and behavior of a physical asset through a bidirectional data connection. This link allows the digital model to reproduce the current state of the physical entity while retaining historical information that supports simulation and immediate problem solving (Grieves and Vickers, 2016; Iliuţă et al., 2024; Singh et al., 2021). Figure 1 illustrates this concept through the example of a hotel receptionist DT, depicting how the employee, together with the resources and physical and social environment in which they operate, generates continuous information that feeds the DT. This data is then processed to support predictive analysis and provide feedback that can enhance employee performance and decision making.

The ability to combine real-time monitoring with predictive analytics has facilitated the adoption of DTs across sectors such as manufacturing, engineering, energy, and healthcare, where they support process visualization and strategic decision making (Iliuţă et al., 2024; Semeraro et al., 2021; Singh et al., 2021). Their versatility also suggests a likely expansion toward biological and human-centered contexts in the coming years (Crespi et al., 2023), providing a conceptual basis for the progressive evolution of DTs toward more personalized digital representations.

Advances in the Internet of Things, artificial intelligence (AI), big data, and cloud computing have accelerated this evolution by transforming static digital replicas into dynamic systems capable of learning from real-time sensing and anticipating future behaviors (Iliuţă et al., 2024; Jeong et al., 2022). In tourism and hospitality, these developments align with the principles of smart tourism, which emphasize pervasive connectivity, data-driven intelligence, and technology-enabled personalization to support more efficient, sustainable, and adaptive services (Buhalis, 2020; Gretzel et al., 2015). Within this context, DTs act as enabling tools that enhance operational efficiency through predictive maintenance, safety monitoring, and energy management, while also contributing to service personalization and improved guest experiences (Buhalis, 2020; Rahmadian et al., 2023).

As DTs move toward human-centered applications, interest has grown in the HDT, a virtual representation of an individual that integrates physiological, behavioral, and task-related data (Lauer-Schmaltz et al., 2024; Lin et al., 2024). Unlike traditional DTs designed for machines or infrastructures (Iliuţă et al., 2024; Singh et al., 2021), HDTs capture and simulate how people act, learn, and adapt in real time (Lin et al., 2024; Yang et al., 2024). These capabilities have positioned HDTs as valuable tools in fields such as healthcare and industry, where they are used to optimize workload management, support decision making, and improve safety in complex environments (Lin et al., 2024; Yang et al., 2024). In tourism and hospitality, where service outcomes depend heavily on interpersonal interaction, emotional labor, and situational adaptation (Buhalis et al., 2019), HDTs provide new opportunities to understand employee behavior and strengthen service experience. Modeling individual and contextual signals makes it possible to anticipate employee performance, identify operational constraints, and analyze how service quality emerges from continuous human–environment interaction (Segura-Cedres et al., 2025). It is also important to distinguish HDTs from service robots or AI-enabled systems, which automate or execute tasks independently (Lu et al., 2019). More broadly, unlike conventional AI-based decision-support or automation-oriented systems that primarily focus on task optimization or the replacement of human judgment, a human-centered Digital Twin is grounded in an employee augmentation perspective. From this viewpoint, the HDT is designed to continuously represent the employee within their operational context, supporting learning, adaptation, and interaction over time by augmenting (rather than substituting) employees’ cognitive and decision-making capabilities (Bankins et al., 2024; Lee et al., 2023; Raisch and Krakowski, 2021). In this study, the term “human-centered” is used to emphasize that the employee constitutes the core of the DT system and conceptualized as an active subject rather than merely a data source. Employees actively contribute data, receive feedback, and use the system as a support tool to enhance their own performance and decision-making within the work context. Rather than referring to participatory or user-centered design methodologies, human-centeredness here reflects the central role of employees as both data contributors and primary beneficiaries of the system. Accordingly, understanding employees’ intentions to use complex systems like human-centered Digital Twins requires a socio-technical lens that accounts for how design and work practices interrelate (Bednar and Welch, 2020).

In this study, the HDT is approached from a human-centered and socio-technical perspective that places the employee within the broader operational environment. Rather than viewing the HDT as a standalone digital copy of the person, the model incorporates personal attributes, behavioral indicators, and contextual elements that shape daily hospitality operations (Buhalis, 2020; Iliuţă et al., 2024; Lin et al., 2024; Singh et al., 2021). This approach preserves the individual focus of the HDT while recognizing that employee experience and performance emerge from ongoing interactions with guests, colleagues, and organizational resources characteristic of tourism services (Buhalis et al., 2019; Segura-Cedres et al., 2025). Recent literature has outlined the technical foundations of HDT development, including processes related to data collection, modeling, and optimization (Lin et al., 2024). Building on this foundation, the present study emphasizes employees as active agents whose perceptions, behaviors, and reactions shape the system’s value. From this viewpoint, an employee-centered HDT becomes an integrative framework for understanding how employees adapt, learn, and respond within data-enabled service environments, providing insights into how their experience influences organizational and guest-related outcomes.

### 2.2 Technology adoption and intention to use in the context of human-centered digital twins

To examine how hospitality employees may respond to a human-centered Digital Twin, it is necessary to consider theoretical perspectives that explain individuals’ adoption and use of information systems. Research on technology adoption has traditionally relied on theoretical frameworks such as the Technology Acceptance Model (TAM) (Davis, 1989) and the Unified Theory of Acceptance and Use of Technology (UTAUT) (Venkatesh et al., 2003), which emphasize the role of users’ cognitive evaluations in shaping behavioral intentions. While these models were originally developed to explain the adoption of relatively stable information technologies, recent research indicates that the core perceptual constructs underlying classical adoption frameworks (such as performance expectations, perceived effort, and trust) remain central to understanding user engagement with more complex and adaptive systems, provided they are carefully contextualized to account for socio-technical dynamics and algorithmic decision support (Bankins et al., 2024; Benbya et al., 2020; Lee et al., 2023). In this study, intention to use (IU) refers to employees’ willingness to employ a human-centered Digital Twin in their daily work routines and is considered a key antecedent of actual adoption behavior (Davis, 1989; Venkatesh et al., 2003). Consistent with the Theory of Planned Behavior, favorable perceptions of a technology increase the likelihood of its use (Ajzen, 1991), reinforcing the relevance of examining IU when studying adoption in emerging socio-technical environments such as human-centered Digital Twins. Consistent with TAM and UTAUT, IU is shaped by employees’ expectations regarding system performance and ease of use, as well as by their trust in the system’s reliability and decision-support capabilities. Prior studies have shown that these perceptual mechanisms remain relevant in tourism and hospitality contexts, particularly when examining employees’ adoption of advanced and data-driven digital systems (Ali et al., 2024; Cimbaljević et al., 2023; Hasni et al., 2022), as well as in other organizational settings characterized by increasing technological complexity (Huang et al., 2023; Sepasgozar et al., 2021). Examining IU is particularly relevant in the context of human-centered Digital Twins, as their effectiveness ultimately depends on employees’ voluntary engagement with systems that generate recommendations and insights influencing work practices and decision making.

### 2.3 Perceptual determinants of intention to use a human-centered digital twin

Based on this theoretical foundation, the present research focuses on “individual perception of the system” with three perceptual variables that influence employees’ intention to use a DT: performance expectancy, effort expectancy, and trust in the system.

Performance expectancy (PE) refers to employees’ beliefs that using the system will improve their job performance. Although PE is a well-established determinant of technology adoption and prior research has consistently identified it as one of the strongest predictors of usage intention (Davis, 1989; Davis et al., 1989; Venkatesh et al., 2003), its meaning becomes conceptually distinct in the context of a HDT.

Unlike conventional information systems that support tasks external to the user, a HDT continuously models, analyzes, and provides feedback on the employee’s own behavior and work processes (Lin et al., 2024). This transforms the adoption decision into a reflexive evaluation, where employees assess whether engaging with a digital representation of themselves will augment their judgment, situational awareness, and decision-making, rather than merely automate or standardize work. Recent research on human-AI collaboration and worker augmentation emphasizes that performance-related expectations are central when technologies directly shape how human work is interpreted and supported (Bankins et al., 2024; Lee et al., 2023; Raisch and Krakowski, 2021).

Consistent with technology acceptance theory, when employees expect such systems to enhance their performance, their intention to use them increases (Davis, 1989; Venkatesh et al., 2003).

Therefore, we propose the following hypothesis:

H1 PE positively influences employees’ intention to use a HDT.

Effort expectancy (EE) refers to employees’ perceptions of how easy it is to learn and interact with a system (Davis, 1989; Venkatesh and Bala, 2008). While ease of use has been consistently shown to influence adoption intentions, its relevance becomes particularly salient in the case of human-centered digital twins.

Unlike conventional information systems, a HDT requires employees to interact with a system that continuously represents, interprets, and feeds back information about their own work behavior (Lin et al., 2024). This shifts effort expectancy beyond simple usability concerns toward perceptions of cognitive load, interaction complexity, and interpretability of system outputs. Research on human-AI interaction highlights that when systems are perceived as complex, opaque, or cognitively demanding, users are less likely to engage with them, even if they recognize their potential value (Chauhan and Jaiswal, 2016; Venkatesh and Bala, 2008).

In frontline hospitality contexts, where employees operate under time pressure and manage simultaneous interpersonal and operational demands (Buhalis et al., 2019), perceived effort becomes a critical factor shaping whether advanced digital systems can be realistically integrated into daily work routines. Consistent with technology acceptance theory, lower perceived effort therefore increases intention to use (Chauhan and Jaiswal, 2016; Davis, 1989). Consequently, we propose the following hypothesis:

H2 EE positively influences employees’ intention to use a HDT.

Trust in the system (TS) reflects employees’ beliefs that a system is reliable, secure, and functions as intended (Gefen et al., 2003; Mcknight et al., 2002; Pavlou, 2003; Reichheld and Schefter, 2000). Although trust is a well-established determinant of technology acceptance, it becomes especially salient for a HDT because such systems rely on continuous data capture and analytics about employees’ own work behaviors and performance. This increases uncertainty regarding how data are processed, interpreted, and used, making trust a necessary condition for employees’ willingness to engage.

Recent evidence in workplace AI research shows that transparency versus opacity affects employees’ appraisals and trust in AI, with transparency increasing trust and reducing threat reactions (Yu et al., 2023). Beyond single studies, recent synthesis work emphasizes that trust/distrust regulates AI diffusion and adoption, reinforcing trust as a key condition for acceptance of AI-enabled systems (Afroogh et al., 2024). In organizational settings, qualitative evidence similarly highlights that trust in AI is crucial for adoption, and that perceptions of risk and uncertainty shape how users develop willingness to adopt and rely on AI (Daly et al., 2025).

Therefore, we propose the following hypothesis:

H3 TS positively influences employees’ intention to use a HDT.

**FIGURE 2:**
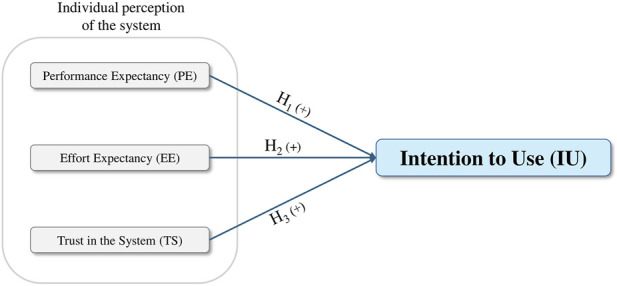
Conceptual model of the perceptual determinants influencing employees’ intention to use a Human-centered Digital Twin.

Figure 2 presents the conceptual model underlying these hypotheses.

Although interest in DT applications is increasing, existing research has focused mainly on technical architecture, operational efficiencies, or customer-facing implementations. Much less attention has been given to the employee as the primary user of a HDT, particularly in hospitality, where frontline staff shape service interactions and determine the feasibility of technological innovations. At present, little empirical evidence exists on how employees perceive a DT designed to mirror their own work activities or which perceptual factors influence their willingness to use such systems. This gap is especially relevant because intention to use is a key antecedent of actual adoption, and the effectiveness of a HDT ultimately depends on the employee’s decision to engage with it. By examining employees’ individual perceptions, including performance expectancy, effort expectancy and trust in the system, this study provides one of the first empirical analyses of intention to use a HDT in hospitality settings, placing employees at the core of digital transformation.

### 2.4 Gamification as a moderating design layer

At the same time, adoption decisions are rarely driven by perceptions alone. How the system is designed and experienced can shape the extent to which these perceptions translate into willingness to use the technology in practice. Prior research suggests that the way a digital system is designed and presented to users can influence how these beliefs are formed and enacted (Davis, 1989; Elshan et al., 2022; Venkatesh and Bala, 2008). One design approach that has received increasing attention is gamification (GAM). GAM is commonly defined as the use of game design elements in non-game contexts (Deterding et al., 2011). In information systems research, GAM is typically discussed as a design approach for creating more “gameful” systems that aim to elicit game-like experiences and motivations to influence user engagement and behavior, rather than as a standalone technology (Koivisto and Hamari, 2019). In fact, systematic reviews show that the empirical effects of GAM are mixed and highly dependent on contextual and design conditions (Hamari et al., 2014; Koivisto and Hamari, 2019). This contingency is particularly salient in organizational settings, where “work gamification” has been conceptualized as an intentional enhancement of performance management systems through, for example, increased access to performance information and more enjoyable task experiences (Cardador et al., 2017). Moreover, empirical research in workplace contexts also indicates that outcomes can vary across employee groups and work-related conditions, reinforcing that GAM effects are not uniform (Gerdenitsch et al., 2020). In the context of a human-centered digital twin, gamification is not merely an engagement mechanism, but a design layer that shapes how employees experience and interpret system feedback about their own work activities.

Because HDTs provide continuous, data-driven representations of employee behavior, gamified elements may alter the cognitive and attentional demands associated with system interaction. Recent research in information systems and human-computer interaction suggests that specific system design characteristics, such as interaction features, feedback mechanisms, and interface structure, can shape how users form and enact their perceptions of overall system evaluation (Elshan et al., 2022).

**FIGURE 3:**
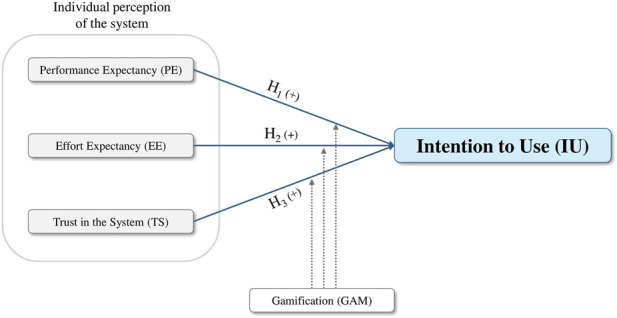
Extended model of the perceptual determinants influencing employees’ intention to use a Human-centered Digital Twin with the moderation role of gamification.

Given the mixed and context-dependent findings reported in prior gamification research, this study does not assume a specific direction for these moderating effects, but rather explores whether and how gamification shapes the translation of perceptual beliefs into intention to use. Accordingly, the extended model explores the moderating role of GAM in the relationships between PE, EE, TS, and intention to use a HDT. Figure 3 presents the extended conceptual model, incorporating GAM as a moderating variable.

## 3 Methodology

### 3.1 Study design and measurement instrument

To examine the individual perceptual factors influencing hospitality employees’ intention to use a HDT, a structured multi-item survey was developed based on previously validated academic scales and previous studies. The items were adapted to reflect the perceptual constructs included in the model: PE, EE, TS, and intention to use. In addition, GAM was included as employees’ perceived presence of game design elements (e.g., challenges, rewards, achievements, and levels) embedded in the interaction with the human-centered digital twin. The construct reflects perceptions of how such elements may facilitate use, support learning, and influence performance in a work context, rather than measuring entertainment or playfulness *per se*. The survey, administered in English, used a seven-point Likert scale ranging from 1 (strongly disagree) to 7 (strongly agree). All constructs were measured using items adapted from previously validated scales in the literature and previous studies. The full list of items and their sources is reported in Supplementary Table S1. The reliability and construct validity of the measurement instrument were assessed through Exploratory Factor Analysis and internal consistency measures, as detailed in Section 3.3, 4.1.

### 3.2 Data collection and sample profile

Data for this study was gathered between March and June 2025 using the Prolific platform, which is widely recognized for its rigorous participant quality controls and transparent recruitment procedures (Palan and Schitter, 2018; Peer et al., 2017). To ensure alignment with the study’s objectives, participation was limited to individuals currently employed in customer-facing roles within European hotels. A total of 258 workers met these criteria and were invited to complete the questionnaire. Of these, 171 respondents finished the survey, and after removing incomplete submissions, the final sample consisted of 141 valid cases.

The characteristics of the participants reflect a diverse cross-section of front-line hospitality employees. As shown in Table 1, a considerable proportion of the sample belonged to the digital native generation (88.7%), and women represented slightly more than half of respondents (55.3%). Professional experience varied considerably: while 39% had fewer than 5 years of industry tenure, nearly one-quarter (24.8%) reported over 10 years of experience. Job roles were primarily concentrated in support positions (38.3%) and front office departments (34.8%), and most employees worked in chain hotels (39%).

**TABLE 1:** Socio-demographic and professional characteristics of respondents.

	N (Number of respondents)	Percentage (%)
Total	141	100
Age		
Digital natives Digital immigrants	125 16	88.7 11.3
Gender		
Men Women Other	62 78 1	44.0 55.3 0.7
Work experience		
Less 5 years From 5 years to 10 years Over 10 years	55 51 35	39.0 36.2 24.8
Job position		
Front office staff Food and beverage staff Recreational and service staff Support staff	49 31 7 54	34.8 21.9 4.9 38.3
Type of hotel		
Airport Boutique Business oriented Chain (e.g., Marriot, Hilton …) Luxury Other	5 14 10 55 20 37	3.5 9.9 7.1 39.0 14.2 26.2

Source: Author’s own.

To facilitate interpretation and avoid excessive fragmentation, individual job titles were grouped into broader categories. Front office staff included roles directly involved in guest reception and relationship management (e.g., receptionists, front desk managers, guest relations officers, and concierge positions). Food and beverage staff comprised employees working in restaurant and bar services, while recreational and service staff included entertainment and animation-related roles, which typically represent smaller staffing groups within hospitality establishments. Support staff encompassed operational roles that enable and sustain service delivery, such as housekeeping/cleaning, maintenance and security, bell services, valet/parking, and administrative support functions linked to front office operations. Although some of these roles involve less direct guest interaction, they are embedded in frontline service processes and contribute to overall service quality and guest experience.

Although demographic variables such as age and gender were collected to contextualize the sample, they were not used for comparative analyses, as the study focuses exclusively on employees’ perceptions toward the technology.

### 3.3 Measurement validation

The measurement model was assessed using an Exploratory Factor Analysis (EFA) to examine the dimensionality of the constructs and evaluate the adequacy of the items included in the study. The analysis was performed in IBM SPSS Statistics v29 using Varimax rotation. Sampling adequacy exceeded recommended thresholds, as indicated by a Kaiser–Meyer–Olkin (KMO) value above 0.70 and a statistically significant Bartlett’s test of sphericity (p < 0.001), confirming that the data were suitable for factor extraction (Williams et al., 2010).

The EFA was conducted on the full set of measurement items, including performance expectancy, effort expectancy, trust in the system, intention to use, and gamification, to validate the factorial structure of all constructs used in both the baseline and the extended models. All items loaded above 0.50 on their corresponding constructs, providing evidence of item relevance and supporting the structural validity of the measurement model (Costello and Osborne, 2005). During the EFA, three items (EE1, EE6 and TS6) were removed because they did not reach the minimum loading threshold or showed inconsistent loading patterns. After their removal, the remaining items exhibited clear and satisfactory loadings on their respective factors. Items measuring GAM loaded on a distinct factor, indicating that this construct was empirically distinguishable from the core perceptual variables included in the model.

Cronbach’s alpha coefficients were used to assess internal consistency reliability. Consistent with established guidelines, all constructs exceed the 0.70 threshold, which is considered indicative of acceptable reliability for exploratory studies (Hair et al., 2019; Tavakol and Dennick, 2011). The gamification scale also demonstrated satisfactory internal consistency, supporting its inclusion as a moderating variable in subsequent analyses. In addition, the cumulative explained variance across the extracted components was greater than 60%, demonstrating that the retained factors captured a substantial proportion of the variance in the dataset and supporting the robustness of the measurement structure (Costello and Osborne, 2005). Descriptive statistics for all items, including means, standard deviations, skewness, and kurtosis, fell within acceptable ranges (±2), suggesting adequate univariate normality for subsequent analyses (Kline, 2023).

After validating the factor structure and confirming reliability, the proposed relationships were tested using multiple linear regression analysis. First, a baseline model was estimated to examine the direct effects of PE, EE, and TS on employees’ intention to use a HDT. Subsequently, an extended model was estimated by including interaction terms to assess the moderating role of GAM on these relationships. Standardized beta coefficients (β) were used to assess the magnitude and direction of the effects, and statistical significance was determined using p-values (Cohen et al., 2013). Model fit was evaluated using the coefficient of determination (*R* ^2^), complemented by the adjusted *R* ^2^ to account for the number of predictors (Mason and Perreault, 1991).

## 4 Results

The results are presented in two stages. First, we report the validation of the measurement model through an EFA, including factor structure, item retention, and reliability indicators. Second, we present the findings from two multiple regression analysis: one to test the study hypotheses regarding the direct effects of PE, EE, and TS on employees’ Intention to Use (IU) a HDT, and another regression analysis for the extended model with the variable GAM as a moderator of PE, EE, and TS.

### 4.1 Measurement model validation

An Exploratory Factor Analysis (EFA) was conducted to assess the dimensionality and psychometric adequacy of the measurement instrument. The analysis used Principal Component Analysis with Varimax rotation in IBM SPSS Statistics v29. Bartlett’s test of sphericity was significant (p < 0.001), and the Kaiser–Meyer–Olkin (KMO) measure exceeded the recommended 0.70 threshold, confirming the suitability of the data for factor extraction (Williams et al., 2010). The EFA included all items corresponding to the constructs examined in the study: PE, EE, TS, IU, and GAM. The resulting factor solution aligned with the theoretical structure of the study and explained more than 60% of the total variance, indicating strong structural validity of the scales (Costello and Osborne, 2005).

Item retention was based on standard criteria from prior literature, including factor loadings above 0.50, conceptual consistency, and absence of cross-loadings (Costello and Osborne, 2005). Three items were removed during this stage due to insufficient loadings or ambiguity. Specifically, EE1 and EE6 showed low or diffuse loadings across multiple factors, while TS6 exhibited inadequate loading in its target construct. After removing these EE1, EE6, and TS6, all remaining indicators loaded cleanly in their respective factors, supporting the expected five-dimensional structure. Supplementary Table S3 presents the final set of items and their rotated loadings.

Internal consistency was assessed using Cronbach’s alpha. All five constructs demonstrated strong reliability, with alpha coefficients exceeding the recommended 0.70 threshold (Hair et al., 2019; Tavakol and Dennick, 2011). In addition, the retained items displayed no issues regarding skewness or kurtosis, with values within the ±2 range, supporting univariate normality assumptions for subsequent analyses (Kline, 2023).

Overall, the EFA provided evidence of the adequacy of the measurement model, providing empirical support for the validity and reliability of the constructs used to examine employees’ perceptions of a human-centered digital twin, as well as the gamification-related design features incorporated in the extended model.

Although structural equation modeling (SEM) is frequently employed in technology adoption research, the primary objective of this study was exploratory and predictive rather than confirmatory. The focus was on identifying key perceptual predictors of intention to use, and on examining whether a salient design feature (gamification) alters how these perceptions translate into intention, rather than testing a complex latent structural model. Therefore, the combination of EFA and multiple linear regression was considered appropriate to validate the measurement structure and estimate predictive relationships based on the observed measures and interaction terms, aligning with the study’s emphasis on early-stage adoption drivers.

### 4.2 Regression analysis and hypothesis testing

To test the proposed hypotheses, a multiple linear regression analysis was conducted with IU as the dependent variable and PE, EE, and TS as predictors. The overall model was statistically significant (F = 58.988, p < 0.001) and accounted for 56.4% of the variance in IU (*R* ^2^ = 0.564, adjusted *R* ^2^ = 0.554), indicating substantial explanatory power for a behavioral model grounded in technology adoption theory.

**FIGURE 4:**
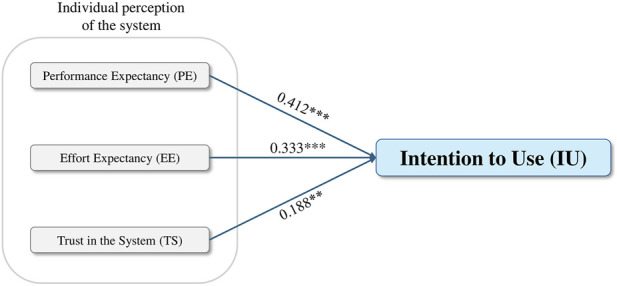
Results of the regression model assessing the determinants of intention to use the human-centered Digital Twin.

The standardized coefficients confirmed that PE was the strongest predictor of employees’ intention to use the human-centered digital twin (β = 0.412, p < 0.001), supporting H_1_. EE also showed a positive and significant effect (β = 0.333, p < 0.001), indicating that perceptions of ease of use meaningfully increase employees’ willingness to adopt the system, thereby supporting H_2_. Finally, TS exerted a smaller yet statistically significant influence on IU (β = 0.188, p = 0.002), providing support for H_3_. Diagnostic tests indicated no multicollinearity issues in the baseline model, with all variance inflation factor (VIF) values below recommended thresholds. A detailed summary of the regression coefficients and hypothesis testing results is presented in Table 2 and Figure 4 illustrates the resulting model with standardized coefficients.

**TABLE 2:** Results of the multiple regression analysis.

Variables	Model		Conclusion
	β coefficient	t (p)	
Performance expectancy (PE)	0.412	5.802 (<0.001) ***	H_1_ supported
Effort expectancy (EE)	0.333	4.479 (<0.001) ***	H_2_ supported
Trust in the system (TS)	0.188	3.109 (0.002) **	H_3_ supported
*R* ^2^	0.564		
Adjusted *R* ^2^	0.554		
F	58.988 (<0.001) ***		

*p < 0.10; **p < 0.05; ***p < 0.01.

Source: Author’s own.

To further explore whether design-related system features influence the relationships identified in the baseline model, an extended regression model was estimated including GAM as a moderating variable. Interaction terms between GAM and each of the three perceptual predictors (PE, EE, and TS) were entered into the regression model, with IU remaining as the dependent variable.

The extended model remained statistically significant and explained a comparable proportion of variance in intention to use (*R* ^2^ = 0.576, adjusted *R* ^2^ = 0.557). The interaction effects revealed that GAM did not significantly moderate the relationships between PE and IU (β = 0.047, p = 0.541), nor between TS and IU (β = 0.016, p = 0.814). However, there is a negative interaction between EE and GAM with marginal significance at 10% level (β = −0.140, p = 0.065). Multicollinearity diagnostics indicated no concerns, with all VIF values well below commonly accepted thresholds.

**FIGURE 5:**
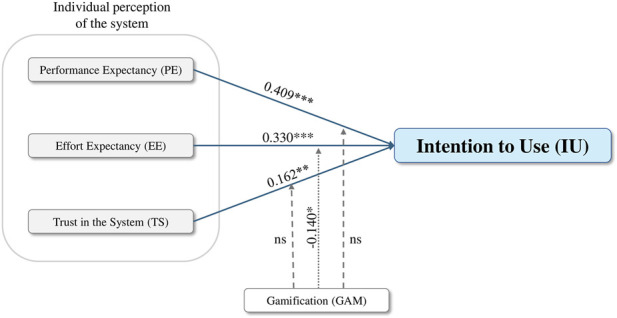
Results of the extended regression model assessing the determinants of intention to use a Human-centered Digital twin with the moderation role of gamification.

This result indicates that the positive effect of EE on employees’ intention to use a HDT decreases as the level of GAM increases. These findings indicate that while perceptions of ease of use remain an important driver of adoption, the presence of gamified design elements alters how employees translate perceived ease into behavioral intention. Moreover, the inclusion of interaction terms resulted in a modest increase in explained variance, suggesting that GAM contributes additional explanatory insight beyond the baseline adoption model. A summary of the extended regression results is presented in Table 3 and Figure 5 illustrates the extended conceptual model including the significant moderating effect.

**TABLE 3:** Results of the extended Multiple Regression Analysis with the moderation role of gamification.

Variables	Model		Conclusion
	β coefficient	t (p)	
Performance expectancy (PE)	0.409	5.274 (<0.001) ***	H_1_ supported
Effort expectancy (EE)	0.330	4.386 (<0.001) ***	H_2_ supported
Trust in the system (TS)	0.162	2.460 (0.015) **	H_3_ supported
Gamification (GAM) x PE	0.047	0.613 (0.541)	Not significant
Gamification (GAM) x EE	−0.140	−1.862 (0.065) *	Marginally significant
Gamification (GAM) x TS	0.016	0.235 (0.814)	Not significant
*R* ^2^	0.576		
Adjusted *R* ^2^	0.557		
F	30.297 (<0.001) ***		

*p < 0.10; **p < 0.05; ***p < 0.01.

Source: Author’s own.

## 5 Discussion

The findings confirm that hospitality employees’ perceptions are central to understanding whether they would be willing to use a human-centered digital twin in their daily work. In the baseline model, all three perceptual determinants show meaningful, positive relationships with intention to use. This reinforces a simple but important point: even when a technology is technically sophisticated and closely connected to the employee’s own behavior, adoption still begins with how employees evaluate the system in practical terms.

Performance expectancy emerges as the most influential driver. In frontline hospitality roles, where service outcomes depend on rapid judgment, emotional labor, and continuous adaptation, employees are especially attentive to whether a system can genuinely help them perform better, reduce uncertainty, or handle demanding interactions more effectively. If the human-centered digital twin is perceived as a tool that supports decisions and improves outcomes, employees appear substantially more inclined to engage with it. From a broader perspective, this finding aligns with the principles of smart tourism, where intelligent systems are expected to support real-time decision making and enhance human performance rather than replace it.

Effort expectancy also plays a strong role in shaping intention. Frontline staff often operate under time pressure while coordinating multiple tasks and social interactions, so usability is not a secondary issue. When the system is perceived as easy to learn and operate, employees can imagine integrating it into routines without disruption. In other words, the more the technology feels intuitive, the more realistic adoption becomes, regardless of how advanced the underlying analytics may be.

Trust in the system is significant as well, although its influence is weaker compared with the other determinants. One plausible explanation is that human-centered digital twins are still unfamiliar in hospitality practice, meaning that respondents may not yet have a clear mental model of what the system would do, what data it would require, or how outputs would be used. Under these conditions, employees may default to evaluating the system through more immediate criteria, such as usefulness and ease of use, while trust becomes less decisive. In addition, the high proportion of digital natives in the sample may matter here. Employees who are more accustomed to digital systems and data-driven tools may be less likely to treat trust as a primary barrier at the intention stage, because baseline comfort with technology reduces perceived uncertainty. This does not mean trust is irrelevant, but rather that its role may become more pronounced in other populations, in later stages of implementation, or once employees experience the system over time and begin to evaluate fairness, transparency, and accountability more concretely.

The extended model adds a complementary layer to this interpretation by showing that design features may shape how core perceptions translate into intention. In this study, gamification does not change the relationships between performance expectancy or trust and intention, suggesting that perceived usefulness and perceived system reliability remain relatively stable drivers regardless of whether gamified elements are present. However, gamification does moderate the relationship between effort expectancy and intention in a way that does not uniformly facilitate adoption: when gamification is higher, the positive influence of effort expectancy becomes weaker. This pattern can be interpreted in several, non-mutually exclusive ways that fit the hospitality work context. First, gamified features may be experienced as an additional interaction layer that employees must learn and manage, especially if they introduce points, challenges, rankings, or progress indicators on top of an already complex service environment. In that case, the system may feel less straightforward, and ease of use becomes harder to translate into genuine willingness to adopt. On the other hand, employees may interpret gamification as a subtle form of control or perceived surveillance, where performance feedback is framed as a game but still functions as monitoring (“gamipulation” (Aguiar-Castillo et al., 2023)). This can create psychological friction, particularly in roles where emotional labor is already high and autonomy is valued. Moreover, gamification may be perceived as encouraging competition among colleagues, which can be counterproductive in service teams that rely on coordination and mutual support.

Taken together, the results suggest that adoption of a human-centered digital twin in hospitality is shaped not only by whether employees believe the system is useful, easy to use, and reliable, but also by how these perceptions are filtered through the system’s interaction design. The baseline model confirms the foundational role of perceptual beliefs in shaping intention to use. At the same time, the extended model shows that design choices, such as gamification, can modify how ease-related perceptions translate into adoption intentions. For technologies that aim to augment rather than replace human work, this finding is particularly relevant. It suggests that employee perceptions constitute the primary pathway to adoption, but that this pathway can be reinforced or disrupted by design and implementation decisions. In this sense, the effectiveness of human-centered digital twins as worker augmentation tools depends not only on their analytical capabilities, but also on whether their design is experienced by employees as supportive, transparent, and aligned with their everyday work practices, rather than as intrusive or demanding.

## 6 Theoretical and practical implications

### 6.1 Theoretical contributions

This study makes several contributions to theory that collectively advance research on digital twins, technology adoption, and human-centered technologies in hospitality contexts.

First, the research contributes to the hospitality and tourism literature by reframing the digital twin as a human-centered and socio-technical system embedded in frontline service work. While digital twins have been predominantly discussed in relation to machines, infrastructures, or operational processes, this study shifts the analytical focus toward frontline employees as the primary users of the technology. By doing so, it extends the digital twin concept into a service work context where performance, interaction, and situational adaptation are inherently human. The findings reinforce the idea that a human-centered digital twin cannot be understood merely as a technological artifact, but rather as a system embedded in everyday work practices, shaped by employees’ perceptions and experiences. By positioning the human-centered digital twin at the level of frontline work, the study also extends smart tourism research beyond destination and customer-centric perspectives, highlighting the role of intelligent systems in augmenting employee capabilities within smart service ecosystems.

Second, the study contributes to technology adoption theory by theoretically contextualizing established perceptual determinants (performance expectancy, effort expectancy, and trust in the system) within a novel and more intimate form of human-technology interaction. Unlike many technologies examined in prior TAM or UTAUT-based studies, a human-centered digital twin is not external to the employee’s tasks but directly mirrors and interprets their own work behaviors and performance. Rather than simply confirming that established adoption relationships hold, the findings suggest that the meaning and salience of these perceptual determinants shift when the technology represents the employee’s own work. In this context, adoption evaluations are shaped not only by usefulness or ease of use, but also by how the system reflects, interprets, and potentially influences employees’ professional activity. This insight extends existing adoption models by demonstrating their applicability and their conceptual transformation into reflexive, employee-centered digital systems.

A further theoretical contribution lies in the integration of system design considerations into the adoption framework through the inclusion of gamification as a moderating variable. Rather than treating adoption as a function of perceptual beliefs alone, the study highlights that design features can shape how these beliefs translate into intention to use. The moderating effect of gamification on the relationship between effort expectancy and intention to use suggests that interaction design choices may alter the perceived simplicity or complexity of advanced systems. This finding adds nuance to the literature on gamification by showing that in professional, human-centered AI systems, gamified elements may also introduce tension by increasing perceived effort or cognitive demands. From a theoretical standpoint, this contribution underscores the importance of moving beyond universal assumptions about gamification and considering its role as a context-dependent design layer within technology adoption models. In doing so, the study contributes to emerging debates on worker augmentation by showing that technologies intended to support employees may generate mixed perceptions depending on how design features interact with task demands, autonomy, and perceived control.

Finally, the study addresses a clear empirical gap in hospitality research by providing evidence on how frontline employees evaluate and respond to a human-centered digital twin. While previous studies have largely focused on technical architectures, operational efficiencies, or customer-facing applications, this research places employees at the core of digital transformation. By linking individual perceptions, system design features, and intention to use within a single analytical framework, the study offers an initial foundation for understanding employee adoption of human-centered digital twins in hospitality settings. Together, these contributions position employee-centered digital twins as a distinct class of human-centered technologies whose adoption dynamics require a contextual reinterpretation of established technology acceptance perspectives.

### 6.2 Practical implications

The findings of this study offer several practical implications for hospitality organizations considering the implementation of human-centered digital twins. First, the results highlight the importance of actively involving frontline employees in the adoption process, not only as end users but as key stakeholders in system implementation. When workers clearly understand how the system can support their daily activities and help them cope with complex service situations, they appear more open to engaging with technology. Communicating concrete benefits, such as improved decision support or greater situational awareness during guest interactions, may therefore play a decisive role in fostering positive attitudes toward adoption.

At the same time, the findings reinforce the central role of perceived ease of use in frontline hospitality contexts. Frontline hospitality employees often operate under time pressure and must juggle multiple tasks simultaneously. In such environments, even advanced systems such as human-centered digital twins need to be perceived as intuitive and manageable and must be designed to integrate seamlessly into existing workflows. Simple interfaces, clear guidance, and targeted training initiatives can help employees integrate the system into their routine without feeling overwhelmed. Designing the technology in a way that supports, rather than disrupts, established work practices is likely to reduce resistance during early stages of implementation.

Trust also emerges as an important, though comparatively less dominant, consideration for adoption. Given that human-centered digital twins rely on the continuous collection and processing of performance-related and contextual data, organizations should be transparent about how this information is used. Transparency about what data are collected, how they are processed, and for what purposes feedback is generated is essential to prevent perceptions of misuse or hidden monitoring. Clear communication and governance practices are therefore critical to sustaining employee acceptance.

The moderating role of gamification adds an additional layer to these practical insights. While gamified elements are often introduced with the intention of enhancing engagement, the results suggest that they can also complicate the user experience. In practice, this means that gamification should be applied cautiously. For some employees, especially in high-pressure service roles, gamified features may be perceived as an additional burden or as a form of implicit control or competition. These perceptions can undermine the perceived ease of use of the system and, consequently, reduce willingness to engage with it. Therefore, managers and system designers should carefully assess whether and how gamification aligns with the specific work context and workforce characteristics. Rather than assuming that gamification will universally enhance acceptance, organizations may benefit from adopting flexible design strategies that allow gamified elements to be adapted, limited, or even avoided depending on employees’ preferences and task demands. This approach is particularly relevant in hospitality settings, where service quality relies heavily on emotional labor, interpersonal skills, and situational judgment.

Overall, the study underscores that the successful implementation of human-centered digital twins depends not only on technological sophistication but on thoughtful alignment with employees’ perceptions, work realities, and interaction needs. By considering not only core adoption drivers but also the effects of design choices such as gamification, hospitality organizations can increase the likelihood that these systems are perceived as supportive tools rather than as sources of additional pressure. In doing so, they move closer to realizing the promise of digital twins as genuinely human-centered technologies within service environments. From a smart tourism perspective, this implies that intelligent systems should not only optimize operations or service outcomes, but also be designed as tools for meaningful worker augmentation, enhancing employees’ ability to act, decide, and adapt in complex service environments.

## 7 Suggestions and limitations

This study is subject to several limitations that should be considered when interpreting the findings and that also open meaningful directions for future research. First, although the sample includes employees from different hotel departments and establishment types, its overall size remains relatively modest. This limits the possibility of conducting more fine-grained analyses across subgroups, such as differences by role, tenure, or organizational context. Future studies based on larger samples could provide a more nuanced understanding of how employees’ perceptions of human-centered digital twins vary across heterogeneous hospitality settings.

A second limitation concerns the generational composition of the sample. Most respondents were digital natives, a group that is generally more familiar with digital technologies and may feel more confident interacting with advanced systems. This characteristic may partially explain why certain perceptual factors, such as trust in the system, played a comparatively weaker role in shaping intention to use. Including a higher proportion of digital immigrants or employees with lower technological exposure would help clarify whether age and digital experience moderate the relevance of different adoption drivers, particularly in relation to effort expectancy and system trust.

The study also adopts an exclusively employee-centered perspective, focusing on frontline staff as the primary users of the human-centered digital twin. While this focus is consistent with the study’s objectives, it necessarily leaves out the viewpoints of managers and supervisors. Future research could benefit from incorporating multiple organizational perspectives to better understand how strategic priorities, managerial expectations, and concerns about monitoring or performance evaluation interact with employees’ perceptions and influence the overall feasibility of implementation.

Finally, the study relies on intention to use as the outcome variable, which is widely recognized as a key antecedent of actual adoption but does not capture real usage behavior. As human-centered digital twins move closer to practical implementation in hospitality contexts, future research should aim to collect behavioral data or adopt longitudinal designs. Such approaches would allow researchers to examine how employees’ perceptions evolve over time and how initial reactions to system features, such as interaction design or gamification elements, translate into sustained use, adaptation, or resistance as the technology becomes embedded in everyday work practices. They would also make it possible to explore how human-centered digital twins function as worker augmentation tools over time, and whether perceptions of support, control, or effort change as employees gain experience with the system.

Beyond these methodological considerations, future research could also explore the broader organizational and sustainability-related implications of human-centered digital twins. In hospitality settings, employee-centered digital technologies may contribute not only to performance and decision support, but also to more sustainable work practices by supporting workload management, reducing cognitive strain, and enhancing employee wellbeing. Examining these longer-term outcomes would help situate human-centered digital twins within broader debates on sustainable human resource management and responsible digital transformation in tourism and hospitality.

## Data Availability

The datasets presented in this article are not readily available because The dataset consists of anonymized survey data collected from adult human participants. Due to confidentiality, data protection, and ethical considerations, the datasets are not publicly available. Data may be shared in anonymized form for academic research purposes only, subject to reasonable request and compliance with data protection regulations. Requests to access the datasets should be directed to Desiree Manzano-Farray, desiree.manzano@ulpgc.es.
