# Supplementary material for: Human-centered digital twins in hospitality: how employee perceptions and system design shape adoption
Source: Front Robot AI. 2026 Apr 1;13:1772854. doi: 10.3389/frobt.2026.1772854 (PMC13078991; doi:10.3389/frobt.2026.1772854)
Supplement: Supplementary file 1 [file Table1.docx]

**Supplementary Table 1.** Survey

| Variables | Items | Authors |
| --- | --- | --- |
| Intention to use (IU) | 1. I am willing to use a digital twin in my role if it becomes available. | (Davis, 1989; Venkatesh et al., 2003, 2012; Venkatesh & Bala, 2008) |
|  | 2. If my organization adopts a digital twin, I would integrate it into my daily activities. |  |
|  | 3. I believe a digital twin would become an essential tool for my work. |  |
|  | 4. I am committed to learning how to use a digital twin effectively if implemented. |  |
|  | 5. I would prioritize incorporating a digital twin into my workflow if it helps improve my job performance. |  |
| Performance Expectancy (PE) | 1. I believe that using a digital twin would allow me to complete my tasks more quickly | (Davis, 1989; Davis et al., 1989; Venkatesh et al., 2003, 2012; Venkatesh & Bala, 2008) |
|  | 2. I believe that a digital twin would increase my work productivity. |  |
|  | 3. I perceive that a digital twin would be a valuable and useful tool for my work |  |
|  | 4. I am confident that the use of a digital twin could open up opportunities to enhance my professional development. |  |
|  | 5. I am confident that a digital twin could be a useful tool to reduce errors in my work. |  |
|  | 6. I believe that a digital twin could facilitate the automation of repetitive tasks. |  |
| Effort Expectancy (EE) | 1. I believe that a user-friendly design would be a key factor to facilitate the use of a digital twin. | (Davis, 1989; Venkatesh et al., 2003, 2012; Venkatesh & Bala, 2008) |
|  | 2. I feel that I would be able to solve technical problems related to the digital twin with adequate support. |  |
|  | 3. I find that integrating a digital twin into my daily tasks would be an easy process |  |
|  | 4. I believe that learning to use a digital twin would be easy for me |  |
|  | 5. I am confident that the operation of a digital twin would be fluent without excessive effort on my part. |  |
|  | 6. I hope that learning to use a digital twin will be intuitive and not time-consuming. |  |
| Trust in the System (TS) | 1. It is important that a digital twin is able to provide reliable and accurate results for its adoption. | (Gefen et al., 2003; McKnight et al., 2002; Parasuraman & Colby, 2015; Wang & Benbasat, 2007) |
|  | 2. A digital twin's guarantee of employee and customer data privacy would be fundamental to its implementation. |  |
|  | 3. A digital twin should enhance my performance and complement my work, instead of replacing it. |  |
|  | 4. The security and suitability of a digital twin to the work environment would be key to its acceptance. |  |
|  | 5. It is important that a digital twin is reliable and does not present frequent problems in its operation. |  |
|  | 6. I am confident that the process of implementing a digital twin would be handled efficiently and safely by the hotel. |  |
| Gamification (GAM) | 1. Including elements such as challenges or rewards could facilitate the use of a digital twin at work. | (Deterding et al., 2011; Hamari, 2017; Hamari et al., 2014) |
|  | 2. I would like the use of a digital twin to include elements such as achievements, badges and levels. |  |
|  | 3. Participating in activities such as friendly competitions or challenges would make the use of a digital twin more motivating. |  |
|  | 4. Including learning dynamics based on challenges or rewards could increase my interest in learning how to use a digital twin. |  |
|  | 5. Using characteristics such as levels, achievements or rewards could improve my performance when interacting with a digital twin. |  |
|  | 6. Elements such as achievements and rewards could help me integrate the use of a digital twin into my work routine faster. |  |

***Note: EE1, EE6, and TS6 were removed during the EFA due to low factor loadings.***

***Source:*** *Authors’ own work*
