# Supplementary material for: Human-centered digital twins in hospitality: how employee perceptions and system design shape adoption
Source: Front Robot AI. 2026 Apr 1;13:1772854. doi: 10.3389/frobt.2026.1772854 (PMC13078991; doi:10.3389/frobt.2026.1772854)
Supplement: Supplementary file 2 [file Table2.docx]

**Supplementary Table 2.** EFA results

| Construct | Item | Loading |
| --- | --- | --- |
| Intention to use (IU) | IU1 | 0.780 |
|  | IU2 | 0.772 |
|  | IU3 | 0.627 |
|  | IU4 | 0.758 |
|  | IU5 | 0.764 |
| Performance Expectancy (PE) | PE1 | 0.863 |
|  | PE2 | 0.859 |
|  | PE3 | 0.865 |
|  | PE4 | 0.763 |
|  | PE5 | 0.820 |
|  | PE6 | 0.790 |
| Effort Expectancy (EE) | EE2 | 0.736 |
|  | EE3 | 0.621 |
|  | EE4 | 0.784 |
|  | EE5 | 0.581 |
| Trust in the System (TS) | TS1 | 0.819 |
|  | TS2 | 0.865 |
|  | TS3 | 0.722 |
|  | TS4 | 0.753 |
|  | TS5 | 0.871 |
| Gamification (GAM) | GAM1 | 0.823 |
|  | GAM2 | 0.896 |
|  | GAM3 | 0.903 |
|  | GAM4 | 0.849 |
|  | GAM5 | 0.886 |
|  | GAM6 | 0.897 |

*Note: EE1, EE6, and TS6 were removed during the EFA due to low factor loadings*

**Source:** Author’s own
